# Identifying a novel ferroptosis-related prognostic score for predicting prognosis in chronic lymphocytic leukemia

**DOI:** 10.3389/fimmu.2022.962000

**Published:** 2022-10-06

**Authors:** Bihui Pan, Yue Li, Zhangdi Xu, Yi Miao, Hua Yin, Yilin Kong, Xinyu Zhang, Jinhua Liang, Yi Xia, Li Wang, Jianyong Li, Jiazhu Wu, Wei Xu

**Affiliations:** ^1^ Department of Hematology, The First Affiliated Hospital of Nanjing Medical University, Jiangsu Province Hospital, Nanjing, China; ^2^ Key Laboratory of Hematology of Nanjing Medical University, Nanjing, China

**Keywords:** chronic lymphocytic leukemia, ferroptosis, prognosis, immune infiltrates, nomogram

## Abstract

**Background:**

Chronic lymphocytic leukemia (CLL) is the most common leukemia in the western world. Although the treatment landscape for CLL is rapidly evolving, there are still some patients who develop drug resistance or disease refractory. Ferroptosis is a type of lipid peroxidation–induced cell death and has been suggested to have prognostic value in several cancers. Our research aims to build a prognostic model to improve risk stratification in CLL patients and facilitate more accurate assessment for clinical management.

**Methods:**

The differentially expressed ferroptosis-related genes (FRGs) in CLL were filtered through univariate Cox regression analysis based on public databases. Least absolute shrinkage and selection operator (LASSO) Cox algorithms were performed to construct a prognostic risk model. CIBERSORT and single-sample gene set enrichment analysis (ssGSEA) were performed to estimate the immune infiltration score and immune-related pathways. A total of 36 CLL patients in our center were enrolled in this study as a validation cohort. Moreover, a nomogram model was established to predict the prognosis.

**Results:**

A total of 15 differentially expressed FRGs with prognostic significance were screened out. After minimizing the potential risk of overfitting, we constructed a novel ferroptosis-related prognostic score (FPS) model with nine FRGs (AKR1C3, BECN1, CAV1, CDKN2A, CXCL2, JDP2, SIRT1, SLC1A5, and SP1) and stratified patients into low- and high-risk groups. Kaplan–Meier analysis showed that patients with high FPS had worse overall survival (OS) (*P*<0.0001) and treatment-free survival (TFS) (*P*<0.0001). ROC curves evaluated the prognostic prediction ability of the FPS model. Additionally, the immune cell types and immune-related pathways were correlated with the risk scores in CLL patients. In the validation cohort, the results confirmed that the high-risk group was related to worse OS (*P*<0.0001), progress-free survival (PFS) (*P*=0.0140), and TFS (*P*=0.0072). In the multivariate analysis, only FPS (*P*=0.011) and CLL-IPI (*P*=0.010) were independent risk indicators for OS. Furthermore, we established a nomogram including FPS and CLL-IPI that could strongly and reliably predict individual prognosis.

**Conclusion:**

A novel FPS model can be used in CLL for prognostic prediction. The model index may also facilitate the development of new clinical ferroptosis-targeted therapies in patients with CLL.

## Introduction

Chronic lymphocytic leukemia (CLL) is characterized by the accumulation of monoclonal B cells in the bone marrow and lymphoid organs (1). It is the most common leukemia in the western world, with an incidence of approximately 4.2 cases per 100,000 people per year (2). Currently, the biological hallmarks of CLL are well recognized as B-cell receptor and Bruton tyrosine kinase signaling (3), as well as resistance to apoptosis mediated by Bcl-2, which has revolutionized CLL clinical management. In the new era of novel therapies, the choice of CLL treatment varies from conventional chemotherapy to highly effective regimens such as anti-CD20 monoclonal antibodies, BTK inhibitors, Bcl-2 inhibitors, and PI3K inhibitors (4–6). The emergence of novel therapies has benefitted many patients, especially in high-risk cases, including those with p53 deletion/mutations who have a poor outcome with conventional chemoimmunotherapy (7). Allogeneic hematopoietic stem-cell transplantation is another choice for selected fit patients, but severe rejection and bone marrow failure make many patients and hematologists hesitant to use this method. Although all these kinds of techniques are available in fighting CLL, some patients still develop disease refractoriness and relapse, which show CLL’s heterogeneity. Therefore, it is important to distinguish high-risk patients from the CLL population during the early stage of disease and apply a more suitable treatment strategy.

Ferroptosis is a distinct form of programmed cell death dependent on iron metabolism. It is quite different from other well-recognized cell death types, such as apoptosis, autophagy, pyroptosis, and necroptosis, in terms of biochemistry, morphology, and genetics. Lipid peroxidation, substantial oxidative stress, and intracellular accumulation of reactive oxygen species (ROSs) are typical characteristics of ferroptosis (8). Dr. Stockwell first introduced the definition of “ferroptosis” in 2012 (9). The action of divalent iron and ester oxygenase catalyzes unsaturated fatty acids that are highly expressed on the cell membrane, leading to lipid peroxidation and inducing ferroptosis (10). Rapidly developing ferroptosis studies suggest the possibility of its prognostic significance in several cancers (11–14). Tumor cells are iron addicted, which means they are more dependent on iron than normal cells. Disorders of iron metabolism can increase the risk of cancer and improve cancer growth (15). Activation of ferroptosis pathways can solve the drug resistance problems of existing chemical agents, providing new therapeutic targets for cancer treatment (16, 17).

To explore the relationship between CLL and ferroptosis, in this study, we filtered differentially expressed ferroptosis-related genes in CLL based on public databases. We built a novel prognostic risk model based on nine ferroptosis-related genes (AKR1C3, BECN1, CAV1, CDKN2A, CXCL2, JDP2, SIRT1, SLC1A5, and SP1). Functional analysis was conducted to elucidate the underlying related biological molecular functions and signaling pathways as well as immune correlations. In addition, we used a CLL patient cohort from our center for validation. Our research aims to build a preliminary prognostic model to improve risk stratification in CLL patients and facilitate more accurate assessment for clinical management.

## Materials and methods

### Database resources

The mRNA sequencing profiles and related clinical information for 151 CLL patients in the GSE22762 dataset were extracted from the Gene Expression Omnibus (GEO) database (https://www.ncbi.nlm.nih.gov/geo/) as the training set. Additionally, the sequencing data of another 188 CLL patients and 32 normal samples from the GSE50006 dataset were used to identify the differentially expressed ferroptosis-related genes. The RNA microarray data were normalized using the “sva” R package. The data from GEO are publicly available.

### Patient and clinical data acquisition

A total of 36 CLL patients diagnosed between 2012 and 2017 at the First Affiliated Hospital of Nanjing Medical University were enrolled in this study as the validation cohort. The diagnosis was established in accordance with the International Workshop on CLL—National Cancer Institute (IWCLL-NCI) criteria. This study was approved by the institutional review board of the First Affiliated Hospital of Nanjing Medical University. Clinical characteristics such as age, sex, Binet stage, B symptoms, lymphocytes, hemoglobin, platelet count, lactate dehydrogenase (LDH), β2-microglobulin (β2-MG), complex karyotype, TP53 disruption, and immunoglobulin heavy-chain gene (IGHV) mutation status were gathered from medical records.

### Sample preparation and RNA-Seq

All the total RNA samples in our center were obtained from the purified CD19^+^ B cells of CLL patients using a CD19^+^ B-Cell Selection Kit (Miltenyi Biotech, Gladbach, Germany). The quality of the extracted RNA was assessed using an RNeasy Micro Kit (QIAGEN, Hilden, Germany). The prepared sequencing libraries were sequenced by a HiSeq X Ten high-throughput sequencing system. The sequences were mapped to hg38 (human genome 38) and aligned using Bowtie and BLAT (the BLAST-like alignment tool). The related fragments per kilobase million (FPKM) values in the validation cohort are listed in **Table S1**.

### Acquisition and functional enrichment analysis of ferroptosis-related gene signatures

A total of 259 FRGs (namely 111 markers, 92 drivers, and 56 suppressors) were obtained from the ferroptosis database FerrDb (http://www.zhounan.org/ferrdb/) (18), as provided in **Table S2**. We then performed univariate Cox regression analysis on FRGs and survival data to identify differential FRGs with prognostic value based on the GSE22762 set. Further screening of the differentially expressed FRGs between CLL and normal samples in the GSE50006 cohort was performed by t-test or Kruskal−Wallis test. A protein−protein interaction (PPI) network was constructed from the STRING database. Moreover, Kyoto Encyclopedia of Genes and Genomes (KEGG) and Gene Ontology (GO) analyses were performed to explore the functional enrichment of the CLL-related FRGs *via* the “clusterprofiler” R package.

### Construction of the risk score prognostic model

Fifteen FRGs considered statistically significant were subsequently incorporated into least absolute contraction and selection operator (LASSO) regression analysis to minimize the potential risk of overfitting with the “glmnet” R package (19). In LASSO analysis, the optimal λ was used to construct the risk score model by 10-fold cross-validation. The risk score of each patient was calculated as follows: risk score = $\sum_{i=1}^{n}{\text{β}}_{\text{i}}*x_{\text{i}}$ . The value of β_i_ represents the coefficients, and χ_i_ represents the gene expression. We divided the patients into low- and high-risk groups based on the optimal risk cutoff. Heatmap plotting of the FRG expression level was performed by the “pheatmap” R package. Dimensionality reduction using principal component analysis (PCA) and t-distributed stochastic neighbor embedding (tSNE) was performed to visualize the distribution of the groups with the “ggbiplot” R package. Receiver operating characteristic (ROC) curves and the corresponding areas under the curve (AUCs) calculated using the “timeROC” R package were used to assess the prognostic ability of the risk score model.

### Evaluation of the relationship between immune cell infiltration and risk stratification

The relative proportions of 22 infiltrating immune cell subtypes were estimated by the CIBERSORT (https://cibersort.stanford.edu/) algorithm. We compared the relative fraction of the immune cell subtypes between the low- and high-risk groups with the Wilcoxon test. Single-sample gene set enrichment analysis (ssGSEA) was performed to estimate the immune infiltration score of 28 immune cell subtypes and 13 immune pathways. The gene sets included in ssGSEA are listed in **Table S3**.

### Establishment of the nomogram models

A nomogram model was established to estimate the probability of 2-, 3-, and 5-year OS by the “rms” R package. The capacity for prognostic prediction was evaluated by the concordance index (C-index).

### Statistical analysis

Data were analyzed by R software (version 4.11) and IBM SPSS statistical software (version 21.0). Continuous variables are presented as the mean ± standard deviation (SD) and were compared by the t-test or Kruskal–Wallis test. Survival curves for the overall survival (OS) time, treatment-free survival (TFS) time, and progression-free survival (PFS) time of the low- and high-risk groups were calculated by the Kaplan–Meier method and compared by the log-rank test with the “survminer” R package. Univariate and multivariate regression analyses were performed to determine the independent risk factors. *P*<0.05 was considered statistically significant.

## Results

### Identification of FRGs in CLL

**Figure 1:**
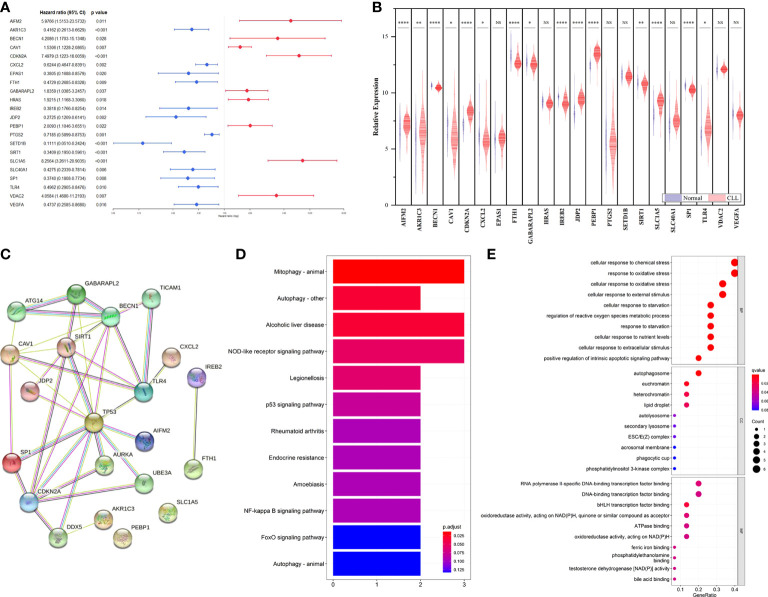
Identification of prognostic FRGs in CLL cohort. **(A)** Forest plot with hazard ratios of the univariate Cox regression analysis showing the FRGs with prognostic value in the GSE22762 dataset. **(B)** The differential expression of FRGs between CLL and normal samples in the GSE50006 dataset. **(C)** PPI network showing the interactions among the 15 FRGs. **(D)** KEGG analyses of the pathways enrichment of the CLL related FRGs. **(E)** GO analyses of the functional enrichment of the CLL related FRGs.

Based on FerrDb, 22 FRGs were found to be related to prognosis in the training cohort (**Figure 1A**). Among them, 13 genes (AKR1C3, CXCL2, EPAS1, FTH1, IREB2, JDP2, PTGS2, SETD1B, SIRT1, SLC1A5, SP1, TLR4, and VEGFA) were correlated with favorable OS, and 9 genes (AIFM2, BECN1, CAV1, CDKN2A, GABARAPL2, HRAS, PEBP1, SLC1A5, and VDAC2) were associated with poor outcomes. We then compared the expression of FRGs that correlated with CLL prognosis between CLL and normal samples, and 15 genes with statistical significance were screened out (**Figure 1B**). Moreover, we explored the FRG correlation with STRING, and the ferroptosis-related gene network indicated potential protein−protein interactions. Interestingly, p53 seemed to be associated with these FRGs (**Figure 1C**). KEGG analysis found that FRGs were enriched in the NOD-like receptor signaling pathway, p53 signaling pathway, NF-κB pathway, and FoxO signaling pathway (**Figure 1D**). GO enrichment analysis revealed that these genes were highly enriched in the biological processes of cellular responses to oxidative stress and chemical stress and DNA-binding transcription factor binding as well as oxidoreductase activity, acting on NAD(P)H in terms of molecular function (**Figure 1E**).

### Establishment and assessment of the novel ferroptosis-related risk score prognostic model

**Figure 2:**
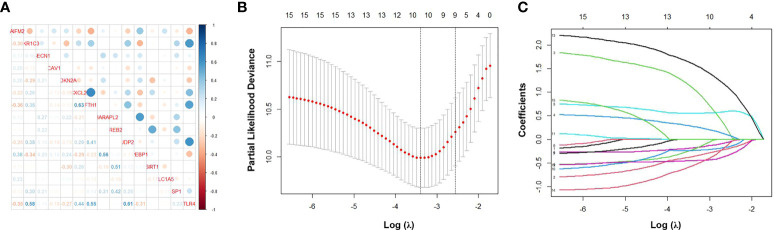
Identification of ferroptosis-related genes signature by LASSO regression algorithm in CLL. **(A)** Correlation analysis of the 15 FRGs. **(B)** Nine prognostic FRGs were identified by LASSO analysis based on optimal weight coefficients (λ). **(C)** The LASSO coefficient profiles of the nine-FRG signature.

**Figure 3:**
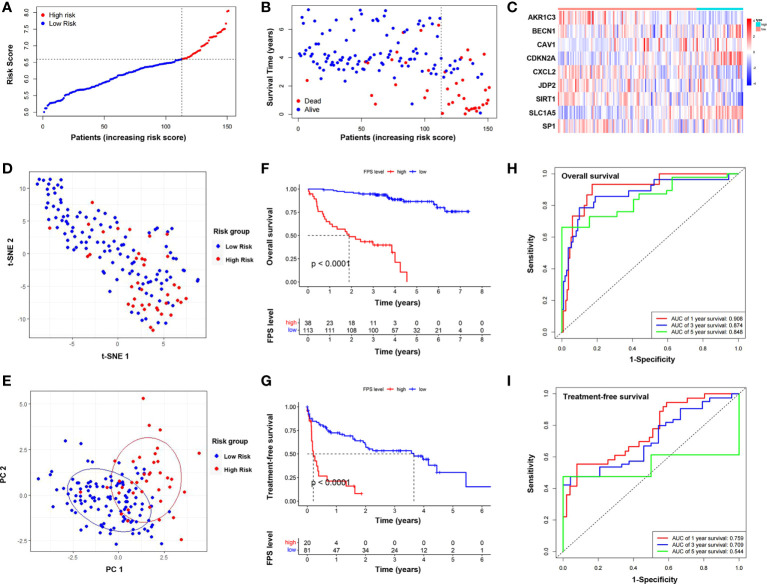
Establishment and assessment of the novel ferroptosis-related gene risk score model. **(A)** The distribution and optimal cutoff value of risk scores in training cohort. **(B)** The distributions of OS status, OS, and risk score. **(C)** Heatmap of the expression levels of the nine selected FRGs. **(D)** t-SNE plot of the CLL cohort. **(E)** PCA plot of the CLL cohort visualizing the distribution of the low- and high-risk groups. Kaplan–Meier survival curves for OS **(F)** and TFS **(G)** of CLL patients stratified by FPS risk score. Time-dependent ROC curves of the risk model for predicting the 1-, 3-, and 5-year OS **(H)** and TFS **(I)**.

We performed correlation analysis of the 15 FRGs, which elucidated the interactions among these genes (**Figure 2A**). Considering the influence of multicollinearity on prediction accuracy, LASSO Cox regression analysis was applied to establish a novel ferroptosis-related prognostic score (FPS) model. Nine prognostic FRGs (AKR1C3, BECN1, CAV1, CDKN2A, CXCL2, JDP2, SIRT1, SLC1A5, and SP1) were identified by LASSO analysis based on optimal weight coefficients (λ) (**Figures 2B, C**). Kaplan−Meier analyses based on the expression of nine genes are shown in **Figure S1**. The risk score model was calculated by the formula FPS = (−0.1930)*AKR1C3 + (0.1751)*BECN1 + (0.0897)*CAV1 + (0.5965)*CDKN2A + (−0.0544)*CXCL2 + (−0.1560)*JDP2 + (−0.3307)*SIRT1 + (1.0947)*SLC1A5 + (−0.1027)*SP1. Based on the optimal cutoff values (cutoff = 6.5965), the CLL patients were divided into two groups: 113 (74.83%) patients in the low-risk group and 38 (25.17%) patients in the high-risk group. As shown in **Figure 3A**, the patients could be stratified into the low- or high-risk group based on the optimal cutoff value. Consistently, the patients with increased risk scores had unfavorable outcomes (**Figure 3B**). The heatmap displays the expression values of the nine genes across the CLL patients with low- or high-risk scores (**Figure 3C**). As shown in **Figures 3D, E**, PCA and tSNE were used to visualize the distributions of different groups according to FPS. The Kaplan–Meier analysis showed that patients with high FPS had a significantly worse OS (*P*<0.0001) and TFS (*P*<0.0001) (**Figures 3F, G**). Moreover, the AUC values of the ROC curves for predicting the 1-, 3-, and 5-year OS were 0.908, 0.874, and 0.848, respectively, and the 1-, 3-, and 5-year TFS were 0.759, 0.709, and 0.544, respectively (**Figures 3H, I**).

### Functional analysis of the two groups and immune relationship with the risk score model

**Figure 4:**
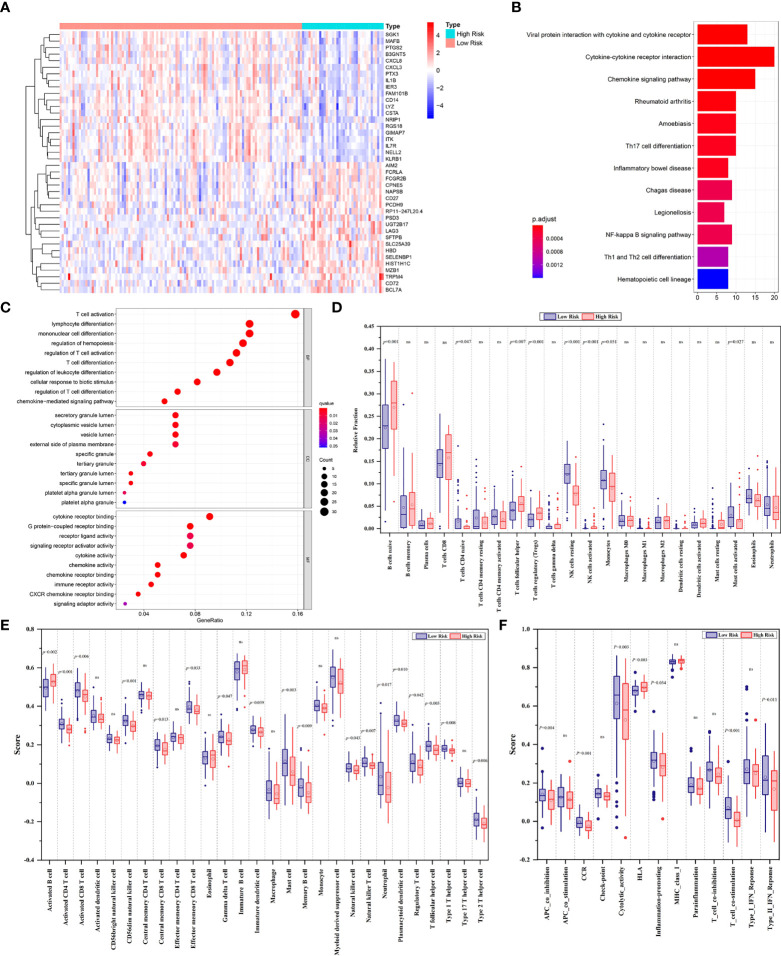
**(A)** The heatmap displaying the differentially expressed genes between the low- and high-risk groups. The representative results of KEGG pathways **(B)** and GO analysis **(C)** of DEGs between two groups. **(D)** Boxplots of the relative fraction of 22 immune cell types between two groups. **(E, F)** The score of immune cell types and immune-related functions using ssGSEA analysis, respectively. ns, no significance.

The heatmap depicted a total of 226 differentially expressed genes (DEGs) between the low- and high-risk groups in line with FPS (**Figure 4A**). All of the DEGs were used to perform KEGG and GO functional enrichment analyses to identify potential biological and molecular functions (**Figures 4B, C**). KEGG analysis showed that several pathways, including Th1- and Th2-cell differentiation, Th17-cell differentiation, the NF-κB signaling pathway, and cytokine−cytokine receptor interactions, were enriched. Notably, DEGs were significantly enriched in T-cell activation, T-cell differentiation, and lymphocyte differentiation by GO functional analysis. We further explored the relationship between the risk scores and immune infiltrates. Considering the uniqueness of the immune microenvironment of hematologic malignancies, we used two algorithms, CIBERSORT and ssGSEA, to estimate the infiltrating immune cell types and related immune pathways. CIBERSORT analysis revealed that the low-risk patients had higher relative fractions of naive CD4^+^ T cells (*P*=0.047), resting natural killer (NK) cells (*P*<0.001), monocytes (*P*=0.051), and activated mast cells (*P*=0.027), while the high-risk patients had higher relative fractions of follicular helper T cells (*P*=0.007), regulatory T cells (*P*<0.001), and activated NK cells (*P*<0.001) (**Figure 4D**). Furthermore, the scores of activated CD4^+^ T cells (*P*<0.001), activated CD8^+^ T cells (*P*=0.006), CD56 dim natural killer cells (*P*<0.001), central memory CD8^+^ T cells (*P*=0.013), effector memory CD8^+^ T cells (*P*=0.033), gamma delta T cells (*P*=0.047), immature dendritic cells (*P*=0.039), mast cells (*P*=0.003), natural killer cells (*P*=0.043), natural killer T cells (*P*=0.007), neutrophils (*P*=0.017), plasmacytoid dendritic cells (*P*=0.010), regulatory T cells (*P*=0.042), follicular helper T cells (*P*=0.003), type 1 T helper cells (*P*=0.008), and type 2 T helper cells (*P*=0.006) were significantly different between the low- and high-risk groups (**Figure 4E**). Additionally, the scores of immune-related pathways, including APC co-inhibition, C–C chemokine receptor (CCR), cytokine activity, inflammation promotion, T-cell costimulation, and type II IFN response, were lower in the high-risk group, while the score of the HLA pathway was higher (**Figure 4F**).

### Validation of the nine-FRG signature prognostic model

**Figure 5:**
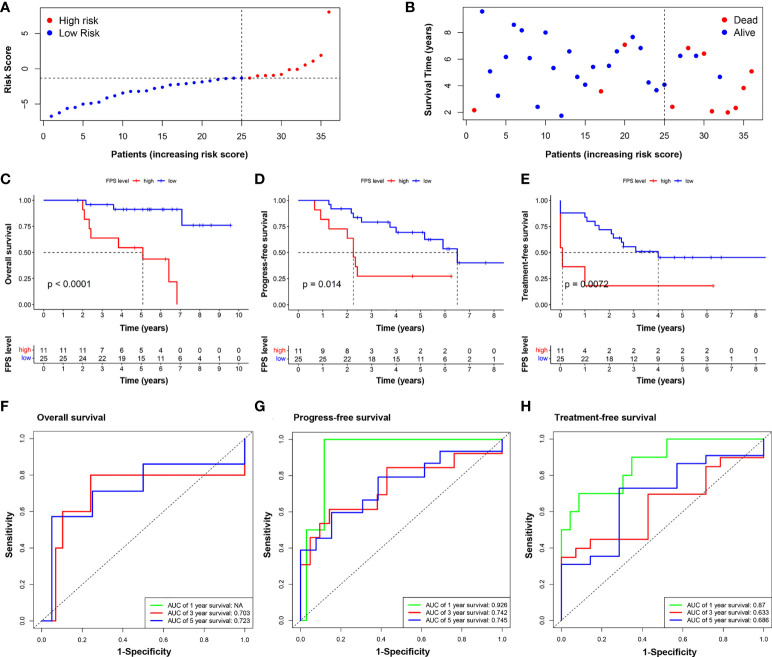
Validation of the nine-FRG signature prognostic model. **(A)** The distribution and the value of risk scores in the validation cohort. **(B)** The distributions of OS status, OS, and risk score. Kaplan–Meier survival curves for OS **(C)**, PFS **(D)**, and TFS **(E)** of CLL patients stratified by FPS risk score. Time-dependent ROC curves of the risk model for predicting the 1-, 3-, and 5-year OS **(F)**, PFS **(G)**, and TFS **(H)**.

**Figure 6:**
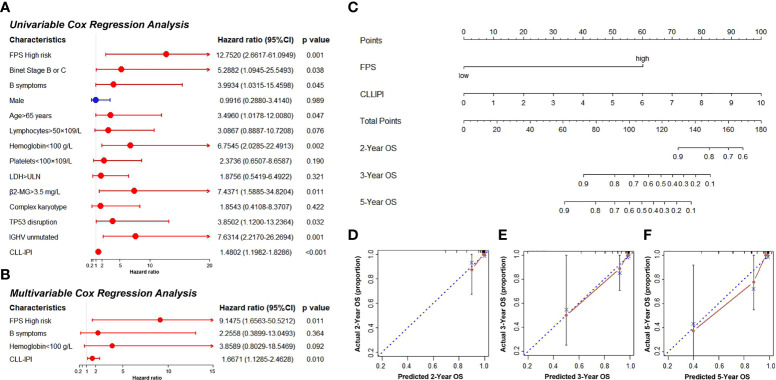
**(A, B)** The univariate and multivariate Cox regression model analyses for assessment of the prognostic value of clinical characteristics and PFS. **(C)** The nomogram model for predicting 2-, 3-, and 5-year OS rate of CLL patients. **(D–F)** The calibration plot analysis to assess the nomogram accuracy for OS prediction at 2, 3, and 5 years.

Thirty-six CLL patients in our center were enrolled in this study as a validation cohort. The clinical characteristics are listed in **Table S4**. We applied the same formula mentioned above to calculate the FPS level based on the RNA sequencing data. Similar to the GEO cohort, the FPS index divided patients into two different risk groups (**Figures 5A, B**). The Kaplan–Meier curves revealed that patients with higher FPS levels had a worse OS (*P*<0.0001), and the AUCs of 3- and 5-year OS were 0.703 and 0.723, respectively (**Figures 5C, F**). The 1-year ROC curve in **Figure 5F** could not be calculated because the OS was 100%. We performed further Kaplan–Meier analyses of PFS and TFS, and the results confirmed that the high-risk group was related to poorer PFS (*P*=0.0140) and TFS (*P*=0.0072) (**Figures 5D, E**). The AUCs of PFS and TFS are specifically depicted in **Figures 5G, H**. In univariate analysis, FPS high risk (*P*=0.001), Binet stage B or C (*P*=0.038), B symptoms (*P*=0.045), age>65 years (*P*=0.047), hemoglobin<100 g/L (*P*=0.002), β2-MG>3.5 mg/L (*P*=0.011), TP53 disruption (*P*=0.032), IGHV unmutated (*P*=0.001), and CLL International Prognosis Index (CLL-IPI) (*P*<0.001) were significantly correlated with inferior OS (**Figure 6A**). Furthermore, we put these factors into a Cox proportional hazards multivariate model. Because Binet stage, age, β2-MG, TP53 disruption, and unmutated IGHV were included in CLL-IPI, CLL-IPI, B symptoms, hemoglobin, and FPS were subsequently integrated to identify independent prognostic factors in the multivariate analysis. The results showed that only FPS (*P*=0.011) and CLL-IPI (*P*=0.010) were independent prognostic indicators for OS (**Figure 6B**).

### Construction and evaluation of nomogram models

Since FPS was an independent predictor in patients with CLL, we constructed a novel prognostic model that incorporated the FPS and CLL-IPI score. The nomogram model was used to predict 2-, 3-, and 5-year OS (**Figure 6C**). The C-index of the nomogram model was 0.903. As shown in **Figures 6D–F**, the calibration plots of the nomogram demonstrated acceptable consistency between the predicted survival rate and the actual survival rate at 2, 3, and 5 years, indicating that the nomogram model could reliably predict the prognosis of CLL patients to some degree.

## Discussion

CLL is a highly heterogenous disease. Forty years ago, Rai and Binet risk stratifications were developed as risk score systems, and they have been used for decades. However, with the rapid progress of treatment, these staging systems became insufficient for clinical practice (20). Then, CLL-IPI was introduced (International CLL-IPI working group, 2016). It included TP53 gene mutation/deletion, IGHV mutation status, serum β2-MG, clinical stage, and age. However, the prognosis of some patients cannot be predicted precisely with CLL-IPI. The progression of disease is related to numerous biological parameters, and disease risk stratification remains a great challenge for hematologists.

Ferroptosis is a nonapoptotic form of cell death depending on iron metabolism that can inhibit tumor growth and increase chemotherapy sensitivity. It is widely reported to be associated with carcinogenesis and cancer prognosis (11, 13, 15). Many small molecular compounds have been proven to target iron metabolism and lipid peroxidation by inducing ferroptosis (21), but the prognostic significance and role of the mRNA expression of ferroptosis-related genes in CLL are currently unclear. In our study, a total of 10 differentially expressed FRGs with statistical prognostic significance were screened out. Interestingly, potential protein−protein interaction analysis indicated that p53 appeared to be the hub gene among the net of these FRGs. p53 is a well-known critical regulator in controlling cell proliferation and survival. Accumulating evidence has revealed that p53 plays an important role in regulating ferroptosis (22). Studies have shown that p53 may play a dual role in the control of ferroptosis, in which it could enhance and suppress it (22). In CLL, the aberration of p53 is frequently associated with disease progression and therapeutic resistance (23). Thus, a further understanding of the impact of p53 on ferroptosis in the context of CLL may provide new therapeutic avenues.

After minimizing the potential risk of overfitting, we established nine ferroptosis-related genes associated with CLL prognosis: AKR1C3, BECN1, CAV1, CDKN2A, CXCL2, JDP2, SIRT1, SLC1A5, and SP1. AKR1C3 is overexpressed in acute myeloid leukemia and T-cell acute lymphoblastic leukemia and confers chemotherapeutic resistance to anthracycline, which is the first-line agent for leukemia treatment (24). AKR1C3 and CXCL2 are ferroptosis-related genes associated with the immune microenvironment and prognosis in breast cancer (25). CXCL2 is a chemokine secreted by monocytes and macrophages and is chemotactic for polymorpho-nuclear leukocytes and hematopoietic stem cells. It is reported to be significantly upregulated in primary CLL patient plasma (26). The addition of CXCL2 enhances CLL cell survival. Chen reported that CXCL2 is a significant ferroptosis-related gene signature that can effectively classify DLBCL into different risk groups in terms of survival rate (14). Ferroptosis is a type of autophagy-dependent cell death. BECN1 influences the onset and progression of autophagy, and its recurrent allelic deletion and expression variation are reported in tumors (27). CAV1 is downregulated after treatment in APL patients and can be used as a potential marker (28). It can inhibit ferroptosis in cancer cells and promote proliferation, migration, and invasion. CDKN2A genetic variation is associated with leukemia incidence and prognosis. CDKN2A and AKR1C1 were established as novel ferroptosis-related signatures that could effectively predict colorectal cancer prognosis (29). JDP2 was identified and validated to be a ferroptosis-related gene signature for predicting survival in cutaneous melanoma (30). SIRT1 and NK-κB are antagonistic in controlling inflammatory processes and energy metabolism (31). The SIRT1–autophagy axis inhibits excess iron-induced ferroptosis of foam cells and subsequently increases IL-1B and IL-18 (32). SLC1A5 is a novel prognostic biomarker that correlates with immune infiltrates in stomach adenocarcinoma *via* ferroptosis. The STAT3–MYC axis promotes the survival of leukemia stem cells by regulating SLC1A5 and oxidative phosphorylation (33). SP1 is reported to be a ferroptosis-related marker in gastric cancer (34). Li Lin showed that inhibiting SP1 could repress ferroptosis of endothelial cells and retard the occurrence of atherosclerosis (35). PKCβII overexpression is related to CLL and the pathogenesis of other B-cell malignancies. Ola-Al-Sanabra et al. first reported that SP1 is linked to this mechanism (36). In summary, AKR1C3, BECN1, CAV1, CDKN2A, CXCL2, JDP2, SIRT1, SLC1A5, and SP1 are related to both ferroptosis and cancer pathogenesis or progression in some way. However, more functional studies are needed to explore the detailed mechanism.

The nine-FRG signature prognostic model can divide the CLL population into high- and low-risk groups. Subsequently, gene function enrichment analysis was performed to reveal the potential mechanism between the two groups. We discovered that DEGs were involved in several pathways, including the IL17 signaling pathway, NK-κB signaling pathway, and regulation of immune system processes. IL 17 is a universal proinflammatory cytokine secreted by a variety of cells, including Th17 cells, innate lymphoid cells (ILCs), NK cells, and γδT cells (37). In CLL, the IL17 level was reported to be increased compared with normal controls and was associated with poor outcome (37). Recent studies have reported that the IL-17 signaling pathway may participate in iron metabolism (38). The NK-κB pathway plays an important role in signal transduction and cell metabolism, promoting proliferation and survival in B-cell malignancies. The NK-κB pathway is constitutively activated in CLL patients and contributes to the pathophysiology of the disease (39). Interestingly, the immunoregulation process, which consists of many cell types, such as B cells, T cells, NK cells, monocytes, and macrophages, is involved in iron metabolism and homeostasis (40). Bioinformatic studies in several cancers have shown that ferroptosis may be implicated in immune infiltrates and pathways (41–43). It has been confirmed that immunotherapy-activated CD8^+^ T cells promote tumor ferroptosis (44). In our study, the high-risk group correlated with an impaired immune response, including low scores of activated CD4^+^ T cells, activated CD8^+^ T cells, and CD56 dim natural killer cells, as well as the decreased activity of APC costimulation, CCR, cytokine activity, T-cell costimulation, inflammation promotion, and type II IFN response. It is speculated that the poor prognostic survival outcomes of the high-risk group may be partially due to immunosuppression.

Our research has some limitations. Due to the less abundant data content of samples in the GEO databases, there was some bias. In addition, our validation cohort had only 36 cases. A prospective and well-designed clinical trial is needed to confirm our findings. Moreover, we also need more functional studies to explore the molecular mechanisms of the role of ferroptosis-related genes in CLL progression.

## Data availability statement

The data of our CLL samples presented in the study are deposited in the DRYAD repository at: https://doi.org/10.5061/dryad.z612jm6fp.

## Ethics statement

This study was reviewed and approved by the institutional review board of the First Affiliated Hospital of Nanjing Medical University. The patients/participants provided their written informed consent to participate in this study. Written informed consent was obtained from the individual(s) for the publication of any potentially identifiable images or data included in this article.

## Author contributions

BP performed data analysis and interpretation. BP and YL drafted the manuscript. YM, JW, ZX, and YX were responsible for RNAseq. HY, YK, XZ, JhL, and LW collected and assembled the clinical data. JyL provided the study design. JW and WX revised the manuscript. All authors contributed to the article and approved the submitted version.

## Funding

This work was supported by the National Natural Science Foundation of China (81770166, 81720108002, 81800192, 82100207); Jiangsu Province’s Medical Elite Programme (ZDRCA2016022); Project of National Key Clinical Specialty, Jiangsu Provincial Special Program of Medical Science (BE2017751), and National Science and Technology Major Project (2018ZX09734007); Nature Science Foundation for Youths of Jiangsu Province (BK20171079, BK20210962); and Young Scholars Fostering Fund of the First Affiliated Hospital of Nanjing Medical University (PY2021026). 

## Conflict of interest

The authors declare that the research was conducted in the absence of any commercial or financial relationships that could be construed as a potential conflict of interest.

## Publisher’s note

All claims expressed in this article are solely those of the authors and do not necessarily represent those of their affiliated organizations, or those of the publisher, the editors and the reviewers. Any product that may be evaluated in this article, or claim that may be made by its manufacturer, is not guaranteed or endorsed by the publisher.
