# Supplementary material for: Identifying a novel ferroptosis-related prognostic score for predicting prognosis in chronic lymphocytic leukemia
Source: Front Immunol. 2022 Oct 6;13:962000. doi: 10.3389/fimmu.2022.962000 (PMC9582233; doi:10.3389/fimmu.2022.962000)
Supplement: Supplementary file 1 [file DataSheet_1.docx]

**Table S1.** 4-FRGs normalized FPKM values in validation cohort.

| **Patients** | **AKR1C3** | **BECN1** | **CAV1** | **CDKN2A** | **CXCL2** | **JDP2** | **SIRT1** | **SLC1A5** | **SP1** |
| --- | --- | --- | --- | --- | --- | --- | --- | --- | --- |
| No01 | 0.0000 | 0.7051 | 0.0000 | 2.3890 | 0.0326 | 3.0179 | 0.3403 | 0.2022 | 0.9668 |
| No02 | 0.0000 | 3.9809 | 0.0418 | 2.4782 | 0.0347 | 4.4514 | 2.5677 | 0.1467 | 16.8791 |
| No03 | 0.0000 | 4.1454 | 0.0000 | 0.9419 | 0.0786 | 2.9850 | 4.1071 | 1.1953 | 17.2467 |
| No04 | 0.0000 | 4.3184 | 0.0000 | 3.2676 | 0.0000 | 3.0217 | 6.8132 | 0.1224 | 17.7680 |
| No05 | 0.0000 | 4.1617 | 0.0000 | 1.6394 | 0.0687 | 1.1758 | 5.7121 | 0.1840 | 16.3795 |
| No06 | 0.0000 | 6.9329 | 0.0000 | 0.5947 | 0.1653 | 4.9488 | 11.2793 | 0.0839 | 20.9849 |
| No07 | 0.0000 | 4.7633 | 0.0067 | 1.4399 | 0.2679 | 1.2169 | 12.5251 | 0.0849 | 21.1551 |
| No08 | 0.0110 | 5.6055 | 0.0000 | 3.1032 | 0.0634 | 2.4864 | 4.6784 | 0.1874 | 11.8956 |
| No09 | 0.0000 | 4.3318 | 0.0071 | 1.9612 | 0.2462 | 3.6426 | 7.4300 | 0.1288 | 11.9892 |
| No10 | 0.0000 | 3.9389 | 0.0000 | 0.6672 | 0.3393 | 6.3959 | 13.6700 | 0.0695 | 12.1033 |
| No11 | 0.0000 | 4.9307 | 0.0060 | 1.4296 | 2.4716 | 4.8092 | 15.6178 | 0.0425 | 11.5851 |
| No12 | 0.0000 | 4.8287 | 0.0119 | 0.9633 | 0.0591 | 0.4540 | 19.7469 | 0.8581 | 24.1704 |
| No13 | 0.0000 | 4.1917 | 0.0058 | 1.2951 | 0.3756 | 1.8080 | 10.4360 | 0.0733 | 19.1102 |
| No14 | 0.0000 | 4.4078 | 0.0000 | 1.9963 | 0.0000 | 2.4897 | 7.2486 | 1.4884 | 22.6204 |
| No15 | 0.0000 | 3.8601 | 0.0051 | 1.9047 | 4.1001 | 2.7814 | 6.7150 | 0.3085 | 5.6751 |
| No16 | 0.0000 | 3.7737 | 0.0556 | 2.3879 | 0.0000 | 2.2003 | 11.0733 | 0.4368 | 17.2350 |
| No17 | 0.0000 | 4.4762 | 0.0075 | 2.3608 | 0.0000 | 4.1857 | 7.4768 | 0.0953 | 14.1277 |
| No18 | 0.0000 | 2.4157 | 0.1306 | 1.2713 | 0.0000 | 5.0772 | 2.2089 | 0.1527 | 7.7828 |
| No19 | 0.0000 | 3.4129 | 0.0000 | 1.6412 | 0.0000 | 1.6970 | 3.4584 | 1.2532 | 9.7778 |
| No20 | 0.0000 | 3.6463 | 0.0000 | 1.8358 | 0.0000 | 2.5502 | 3.7939 | 8.4546 | 12.6021 |
| No21 | 0.0000 | 2.9248 | 0.0209 | 2.8109 | 0.0347 | 2.0152 | 2.5912 | 0.0586 | 11.3061 |
| No22 | 0.0000 | 3.5073 | 0.0112 | 3.1483 | 0.0000 | 2.0509 | 3.4155 | 0.1095 | 19.0577 |
| No23 | 0.0000 | 4.1125 | 0.0063 | 1.8025 | 0.3773 | 2.5477 | 5.8417 | 0.4429 | 18.4465 |
| No24 | 0.1566 | 4.9578 | 0.1293 | 2.1984 | 0.8694 | 3.8420 | 8.1463 | 0.1179 | 15.2159 |
| No25 | 0.0095 | 7.2887 | 0.0386 | 2.1946 | 0.0000 | 2.3562 | 8.8098 | 0.0464 | 14.1917 |
| No26 | 0.0736 | 4.4619 | 0.0000 | 2.1913 | 1.9314 | 5.2594 | 7.9475 | 0.0398 | 13.1004 |
| No27 | 0.1932 | 5.4361 | 0.0056 | 1.6594 | 0.1390 | 1.5454 | 6.3267 | 1.0576 | 19.9430 |
| No28 | 0.0182 | 5.0192 | 0.0736 | 2.9136 | 0.1833 | 3.2006 | 6.0656 | 0.0516 | 15.0682 |
| No29 | 0.2483 | 5.0969 | 0.0053 | 0.8761 | 0.3441 | 4.6969 | 10.3705 | 0.6414 | 16.6634 |
| No30 | 0.2213 | 4.4716 | 0.0000 | 0.8112 | 0.1456 | 1.6237 | 6.2435 | 0.1487 | 24.2963 |
| No31 | 0.0275 | 3.7302 | 0.1219 | 1.0598 | 0.0000 | 2.3965 | 13.6627 | 0.0000 | 25.7398 |
| No32 | 0.1345 | 6.0725 | 0.4749 | 4.2703 | 0.0645 | 2.6955 | 2.1909 | 0.0303 | 5.9284 |
| No33 | 0.0000 | 5.1871 | 0.0425 | 1.8339 | 0.0000 | 4.7505 | 10.6785 | 0.0224 | 25.8060 |
| No34 | 0.2876 | 6.0824 | 0.0332 | 0.8272 | 0.7452 | 4.3608 | 7.8670 | 0.2255 | 15.8144 |
| No35 | 0.0000 | 5.0132 | 0.3958 | 0.6663 | 1.1398 | 2.6521 | 8.6759 | 1.7619 | 11.7978 |
| No36 | 0.1785 | 5.4118 | 0.0000 | 1.5806 | 0.1371 | 1.5183 | 8.6281 | 0.0000 | 18.3267 |

Abbreviations: FRG, ferroptosis-related genes; FPKM, fragments per kilobase million.

**Table S2.** List of the ferroptosis-related Genes enrolled in this study.

| **Classification** | **Ferroptosis-related Genes** |
| --- | --- |
| Ferroptosis Marker | ACSF2 AGPAT3 ALB ALOX12 ALOX15 ALOX5 ANGPTL7 ARRDC3 ASNS ATF3 ATF4 ATP5MC3 ATP6V1G2 AURKA BLOC1S5-TXNDC5 BNIP3 CAPG CBS CEBPG CHAC1 CXCL2 DDIT3 DDIT4 DRD4 DRD5 DUSP1 EIF2AK4 EIF2S1 ELAVL1 FTH1 FTL GABPB1 GDF15 GLUT13 GPT2 GPX2 GPX4 HAMP HBA1 HERPUD1 HIC1 HMGB1 HMOX1 HNF4A HSD17B11 HSPB1 IL33 IL6 IREB2 JDP2 KIM-1 KLHL24 LOC284561 LOC390705 LURAP1L MAFG MAP3K5 MAPK14 MIR30B MIR4715 MT3 NCF2 NFE2L2 NGB NNMT NOS2 OXSR1 PCK2 PLIN4 PRDX1 PSAT1 PTGS2 RELA RGS4 RIPK1 RPL8 RRM2 SELENOS SESN2 SETD1B SLC1A4 SLC2A1 SLC2A12 SLC2A14 SLC2A3 SLC2A6 SLC2A8 SLC3A2 SLC40A1 SLC7A11 SLC7A5 SNORA16A SP1 SRXN1 STEAP3 STMN1 TF TFAP2C TFRC TRIB3 TSC22D3 TUBE1 TXNIP TXNRD1 UBC VEGFA VLDLR XBP1 YWHAE ZFP69B ZNF419 |
| Ferroptosis Driver | ABCC1 ACO1 ACSL4 ACVR1B ALOX12B ALOX15B ALOXE3 ANO6 ATG13 ATG16L1 ATG3 ATG4D ATG5 ATG7 ATM BACH1 BAP1 BECN1 BID CARS1 CDKN2A CDO1 CS CYBB DNAJB6 DPP4 DUOX1 DUOX2 EGFR EGLN2 EMC2 EPAS1 FBXW7 FLT3 G6PD G6PDX GABARAPL1 GABARAPL2 GLS2 GOT1 HIF1A HILPDA HRAS IDH1 IFNG KEAP1 KRAS LINC00472 LONP1 LPCAT3 LPIN1 MAP1LC3A MAPK1 MAPK3 MAPK8 MAPK9 MIOX MIR6852 MTDH MYB NCOA4 NOX1 NOX3 NOX4 NOX5 NRAS PANX1 PEBP1 PGD PHKG2 PIK3CA PRKAA1 PRKAA2 SAT1 SCP2 SIRT1 SLC1A5 SLC38A1 SNX4 SOCS1 TAZ TFR2 TGFBR1 TLR4 TNFAIP3 ULK1 ULK2 VDAC2 WIPI1 WIPI2 YY1AP1 ZEB1 |
| Ferroptosis Supressor | ACSL3 AIFM2 AKR1C1 AKR1C2 AKR1C3 ARNTL BRD4 CA9 CAV1 CD44 CDKN1A CHMP5 CHMP6 CISD1 CISD2 ENPP2 FADS2 FANCD2 Fer1HCH FH FTMT GCH1 GCLC HELLS HSF1 HSPA5 ISCU JUN LAMP2 LINC00336 MIR137 MIR17 MIR212 MIR9-1 MIR9-2 MIR9-3 MT1G MTOR MUC1 NF2 NFS1 NQO1 OTUB1 PLIN2 PML PRDX6 PROM2 RB1 SCD SQSTM1 SRC STAT3 TMBIM4 TP53 TP63 ZFP36 |

**Table S3.** List of the immune infiltration geneset for ssGSEA.

| **Immune Cells** | **Genes** |
| --- | --- |
| Activated B cell | ADAM28 CD180 CD79B BLK CD19 MS4A1 TNFRSF17 IGHM GNG7 MICAL3 SPIB HLA-DOB IGKC PNOC FCRL2 BACH2 CR2 TCL1A AKNA ARHGAP25 CCL21 CD27 CD38 CLEC17A CLEC9A CLECL1 |
| Activated CD4 T cell | AIM2 BIRC3 BRIP1 CCL20 CCL4 CCL5 CCNB1 CCR7 DUSP2 ESCO2 ETS1 EXO1 EXOC6 IARS ITK KIF11 KNTC1 NUF2 PRC1 PSAT1 RGS1 RTKN2 SAMSN1 SELL TRAT1 |
| Activated CD8 T cell | DRM1 AHSA1 C1GALT1C1 CCT6B CD37 CD3D CD3E CD3G CD69 CD8A CETN3 CSE1L GEMIN6 GNLY GPT2 GZMA GZMH GZMK IL2RB LCK MPZL1 NKG7 PIK3IP1 PTRH2 TIMM13 ZAP70 |
| Activated dendritic cell | ABCD1 C1QC CAPG CCL3L3 CD207 CD302 ATP5B ATP5L ATP6V1A BCL2L1 C1QB SNURF SPCS3 CCNA1 CEACAM8 NOS2 SRA1 TNFRSF6B TREM1 TREML1 RHOA SLC25A37 TNFSF14 TREML4 VNN2 XPO6 CLEC4C TNFAIP2 UBD ACTR3 RAB1A SLA HLA-DQA2 SIGLEC5 SLAMF9 |
| CD56bright natural killer cell | ABAT C11orf75 C5orf15 CDHR1 DCAF12 DYNLL1 GPR137B HCP5 HDGFRP2 KRT86 MLST8 ELMOD3 ENTPD5 FAM119A FAM179A CLIC2 COX7A2L CREB3L4 CSF1 CSNK2A2 CSTA CSTB CTPS CTSD FST GATA2 GMPR HDC HEY1 HOXA1 HS2ST1 HS3ST1 BCL11B CDH3 MYL6B NAA16 ClQA ClQB CYP27B1 EIF3M |
| CD56dim natural killer cell | CYP27A1 DDX55 DYRK2 RPL37A NOTCH3 AKR7A3 GPRC5C GRIN1 HLA-E PORCN PSMC4 UPP1 IL21R KIR2DS1 KIR2DS2 KIR2DS5 |
| Central memory CD4 T cell | ABHD3 AHNAK ANXA2P2 AQP3 ATHL1 BMI1 BZW2 CD63 COL4A1 CYLD ELMO2 FYN GLIPR1 GSS IFITM2 ITGB1 ITGB2 KLF5 LSP1 NDUFB9 PKM2 SFXN3 SIRPG SMAD4 STX4 TRADD VIM XRCC6 |
| Central memory CD8 T cell | ACTN4 ADAM12 ADCY9 F13A1 FCER1G FCGR3B FGF7 FKBP4 GLUD1 GM2A GUSB IL1RN NOL11 NTRK1 RARA RNF128 SIGLEC1 TNFRSF11A TOX4 UBA52 ULBP1 |
| Effector memeory CD4 T cell | ATM CASP3 CASQ1 CD300E DARS DOCK9 EXOSC9 EZH2 GDE1 IL34 NCOA4 NEFL PDGFRL PTGS1 REPS1 SCG2 SDPR SIGLEC14 SIGLEC6 TAL1 TFEC TIPIN TPK1 UQCRB USP9Y WIPF1 ZCRB1 |
| Effector memeory CD8 T cell | ACAP1 APOL3 ARHGAP10 ATP10D C3AR1 CCR5 CD160 CD55 CFLAR CMKLR1 DAPP1 FCRL6 FLT3LG GZMM HAPLN3 HLA-DMB HLA-DPA1 HLA-DPB1 IFI16 LIME1 LTK NFKBIA SETD7 SIK1 TRIB23 TMBIM4 TP53 TP63 ZFP36 |
| Eosinophil | GIPR KRT18P50 LRMP FOSB RRP12 GPR183 NR4A3 ST3GAL6 DEPDC5 PDE6C PKD2L2 GPR65 IL5RA P2RY14 DACH1 DAPK2 EMR3 |
| Gamma delta T cell | ACP5 AQP9 BTN3A2 C1orf54 CARD8 CCL18 CD209 CD33 CD36 CDK5 IL10RB KLRF1 LGALS1 MAPK7 KLHL7 KRT80 LAMC1 LCORL LMNB1 MEIS3P1 MPL FABP1 FABP5 FADD MFAP3L MINPP1 RPS24 RPS7 RPS9 DBNL CCL13 |
| Immature B cell | CD22 CYBB FAM129C FCRL1 FCRL3 FCRL5 FCRLA HDAC9 HLA-DQA1 HVCN1 KIAA0226 NCF1 NCF1B P2RY10 SP100 TXNIP STAP1 TAGAP ZCCHC2 |
| Immature dendritic cell | ACADM AHCYL1 ALDH1A2 ALDH3A2 ALDH9A1 ALOX15 AMT ARL1 ATIC ATP5A1 CAPZA1 LILRA5 RDX RRAGD TACSTD2 INPP5F RAB38 PLAU CSF3R SLC18A2 AMPD2 CLTB C1orf162 |
| Macrophage | AIF1 CCL1 CCL14 CCL23 CCL26 CD300LB CNR1 CNR2 EIF1 EIF4A1 FPR1 FPR2 FRAT2 GPR27 GPR77 RNASE2 MS4A2 BASP1 IGSF6 HK3 VNN1 FES NPL FZD2 FAM198B HNMT SLC15A3 CD4 TXNDC3 FRMD4A CRYBB1 HRH1 WNT5B |
| Mast cell | ADAMTS3 CPA3 CMA1 CTSG ARHGAP15 CPM FCN1 FTL HSPA6 ITGA9 RNASE3 S100A4 SIGLEC8 SLC6A4 PTGS2 EGR3 PILRA |
| Memory B cell | AICDA CCNA2 CDKN3 CLCN5 ENPP1 FCER1A FCRL4 MYC RUNX2 SORL1 SOX5 STAT5A STAT5B TLR9 |
| Monocyte | ASGR2 CFP ASGR1 CD1D UPK3A ACTG1 ANXA5 ATP6V1B2 CFL1 DAZAP2 CTBS EMR4P HIVEP2 MARCKSL1 MBP MMP15 PNPLA6 TMBIM6 PQBP1 TEX264 IKZF1 |
| Myeloid derived suppressor cell | CCR2 CD14 CD2 CD86 CXCR4 FCGR2A FCGR2B FCGR3A FERMT3 GPSM3 IL18BP IL4R ITGAL ITGAM PARVG PSAP PTGER2 PTGES2 S100A8 S100A9 |
| Natural killer cell | AKT3 AXL BST2 CDH2 CRTAM CSF2RA CTSZ CXCL1 CYTH1 DAXX DGKH DLL4 DPYD ERBB3 F11R FAM27A FAM49A FASLG FCGR1A FN1 FSTL1 FUCA1 GBP3 GLS2 GRB2 LST1 BCL2 CDC5L FGF18 FUT5 FZR1 GAGE2 IGFBP5 KANK2 LDB3 |
| Natural killer T cell | BTN2A2 CD101 CD109 CNPY3 CNPY4 CREB1 CRTC2 CRTC3 CSF2 KLRC1 FUT4 ICAM2 IL32 LAMP2 LILRB5 KLRG1 HSPA4 HSPB6 ISM2 ITIH2 KDM4C KIR2DS4 KIRREL3 SDCBP NFATC2IP MICB KIR2DL1 KIR2DL3 KIR3DL1 KIR3DL2 NCR1 FOSL1 TSLP SLC7A7 SPP1 TREM2 UBASH3A YBX2 CCDC88A CLEC1A THBD PDPN VCAM1 EMR1 |
| Neutrophil | CREB5 CDA CHST15 S100A12 APOBEC3A CASP5 MMP25 HAL C1orf183 FFAR2 MAK CXCR1 STEAP4 MGAM BTNL8 CXCR2 TNFRSF10C VNN3 |
| Plasmacytoid dendritic cell | CBX6 DAB2 DDX17 HIGD1A IDH3A IL3RA MAGED1 NUCB2 OFD1 OGT PDIA4 SERTAD2 SIRPA TMED2 ENG FCAR IGF1 ITGA2B GABARAP GPX1 KRT23 PROK2 RALB RETNLB RNF141 SEC14L1 SEPX1 EMP3 CD300LF ABTB1 KLHL21 PHRF1 |
| Regulatory T cell | CCL3L1 CD72 CLEC5A FOXP3 ITGA4 L1CAM LIPA LRP1 LRRC42 MARCO MMP12 MNDA MRC1 MS4A6A PELO PLEK PRSS23 PTGIR ST8SIA4 STAB1 |
| T follicular helper cell | B3GAT1 CDK5R1 PDCD1 BCL6 CD200 CD83 CD84 FGF2 GPR18 CEBPA CECR1 CLEC10A CLEC4A CSF1R CTSS DMN DPP4 LRRC32 MC5R MICA NCAM1 NCR2 NRP1 PDCD1LG2 PDCD6 PRDX1 RAE1 RAET1E SIGLEC7 SIGLEC9 TYRO3 CHST12 CLIC3 IVNS1ABP KIR2DL2 LGMN |
| Type 1 T helper cell | CD70 TBX21 ADAM8 AHCYL2 ALCAM B3GALNT1 BBS12 BST1 CD151 CD47 CD48 CD52 CD53 CD59 CD6 CD68 CD7 CD96 CFHR3 CHRM3 CLEC7A COL23A1 COL4A4 COL5A3 DAB1 DLEU7 DOC2B EMP1 F12 FURIN GAB3 GATM GFPT2 GPR25 GREM2 HAVCR1 HSD11B1 HUNK IGF2 RCSD1 RYR1 SAV1 SELE SELP SH3KBP1 SIT1 SLC35B3 SIGLEC10 SKAP1 THUMPD2 TIGIT ZEB2 ENC1 FAM134B FBXO30 FCGR2C STAC LTC4S MAN1B1 MDH1 MMD RGS16 IL12A P2RX5 CD97 ITGB4 ICAM3 METRNL TNFRSF1A IRF1 HTR2B CALD1 MOCOS TRAF3IP2 TLR8 TRAF1 DUSP14 |
| Type 2 T helper cell | ASB2 CSRP2 DAPK1 DLC1 DNAJC12 DUSP6 GNAI1 LAMP3 NRP2 OSBPL1A PDE4B PHLDA1 PLA2G4A RAB27B RBMS3 RNF125 TMPRSS3 GATA3 BIRC5 CDC25C CDC7 CENPF CXCR6 DHFR EVI5 GSTA4 HELLS IL26 LAIR2 |
| **Immune Pathways** | **Genes** |
| APC_co_inhibition | 10orf54 CD274 LGALS9 PDCD1LG2 PVRL3 |
| APC_co_stimulation | CD40 CD58 CD70 ICOSLG SLAMF1 TNFSF14 TNFSF15 TNFSF18 TNFSF4 TNFSF8 TNFSF9 |
| CCR | BMP1 BMP10 BMP15 BMP2 BMP2K BMP3 BMP4 BMP5 BMP6 BMP7 BMP8A BMP8B BMPER BMPR1A BMPR1B BMPR2 CCL1 CCL11 CCL13 CCL14 CCL15 CCL15-CCL14 CCL16 CCL17 CCL18 CCL19 CCL2 CCL20 CCL21 CCL22 CCL23 CCL24 CCL25 CCL26 CCL27 CCL28 CCL3 CCL3L1 CCL3L3 CCL4 CCL4L1 CCL5 CCL7 CCL8 CCR1 CCR10 CCR2 CCR3 CCR4 CCR5 CCR6 CCR7 CCR8 CCR9 CCRL2 CCRN4L CSF1 CSF1R CSF2 CSF2RA CSF2RB CSF3 CSF3R CX3CL1 CX3CR1 CXCL1 CXCL10 CXCL11 CXCL12 CXCL13 CXCL14 CXCL16 CXCL17 CXCL2 CXCL3 CXCL5 CXCL6 CXCL8 CXCL9 CXCR1 CXCR2 CXCR2P1 CXCR3 CXCR4 CXCR5 CXCR6 EPO EPOR IFNA1 IFNA10 IFNA13 IFNA14 IFNA16 IFNA17 IFNA2 IFNA21 IFNA4 IFNA5 IFNA6 IFNA7 IFNA8 IFNAR1 IFNAR2 IFNB1 IFNE IFNG IFNGR1 IFNGR2 IFNK IFNL1 IFNL2 IFNL3 IFNLR1 IFNW1 IL10 IL10RA IL10RB IL11 IL11RA IL12A IL12B IL12RB1 IL12RB2 IL13 IL13RA1 IL13RA2 IL15 IL15RA IL16 IL17A IL17B IL17C IL17D IL17F IL17RA IL17RB IL17RC IL17RD IL17RE IL17REL IL18 IL18BP IL18R1 IL18RAP IL19 IL1A IL1B IL1F10 IL1R1 IL1R2 IL1RAP IL1RAPL1 IL1RAPL2 IL1RL1 IL1RL2 IL1RN IL2 IL20 IL20RA IL20RB IL21 IL21R IL22 IL22RA1 IL22RA2 IL23A IL23R IL24 IL25 IL26 IL27 IL27RA IL2RA IL2RB IL2RG IL3 IL31 IL31RA IL32 IL33 IL34 IL36A IL36B IL36G IL36RN IL37 IL3RA IL4 IL4I1 IL4R IL5 IL5RA IL6 IL6R IL6ST IL7 IL7R IL9 IL9R ILDR1 ILDR2 ILF2 ILF3 ILK ILKAP ILVBL TGFA TGFB1 TGFB1I1 TGFB2 TGFB3 TGFBI TGFBR1 TGFBR2 TGFBR3 TGFBRAP1 TNF TNFAIP1 TNFAIP2 TNFAIP3 TNFAIP6 TNFAIP8 TNFAIP8L1 TNFAIP8L2 TNFAIP8L3 TNFRSF10A TNFRSF10B TNFRSF10C TNFRSF10D TNFRSF11A TNFRSF11B TNFRSF12A TNFRSF13B TNFRSF13C TNFRSF14 TNFRSF17 TNFRSF18 TNFRSF19 TNFRSF1A TNFRSF1B TNFRSF21 TNFRSF25 TNFRSF4 TNFRSF6B TNFRSF8 TNFRSF9 TNFSF10 TNFSF11 TNFSF12 TNFSF12-TNFSF13 TNFSF13 TNFSF13B TNFSF14 TNFSF15 TNFSF18 TNFSF4 TNFSF8 TNFSF9 TPO XCL1 XCL2 |
| Check-point | ADORA2A BTLA BTNL2 C10orf54 CD160 CD200 CD200R1 CD244 CD27 CD274 CD276 CD28 CD40 CD40LG CD44 CD48 CD70 CD80 CD86 CTLA4 HAVCR2 HHLA2 ICOS ICOSLG IDO1 IDO2 KIR3DL1 LAG3 LAIR1 LGALS9 NRP1 PDCD1 PDCD1LG2 TIGIT TMIGD2 TNFRSF14 TNFRSF18 TNFRSF25 TNFRSF4 TNFRSF8 TNFRSF9 TNFSF14 TNFSF15 TNFSF18 TNFSF4 TNFSF9 VTCN1 |
| Cytolytic_activity | GZMA PRF1 |
| HLA | HLA-A HLA-B HLA-C HLA-DMA HLA-DMB HLA-DOA HLA-DOB HLA-DPA1 HLA-DPB1 HLA-DPB2 HLA-DQA1 HLA-DQA2 HLA-DQB1 HLA-DQB2 HLA-DRA HLA-DRB1 HLA-DRB5 HLA-DRB6 HLA-E HLA-F HLA-G HLA-H HLA-J HLA-L |
| Inflammation-promoting | CCL5 CD19 CD8B CXCL10 CXCL13 CXCL9 GNLY GZMB IFNG IL12A IL12B IRF1 PRF1 STAT1 TBX21 |
| MHC_class_I | B2M HLA-A TAP1 |
| Parainflammation | AIM2 ANXA1 BLNK BST2 CCND1 CD14 CD276 CD44 CXCL10 CXCL9 HMOX1 ICAM1 IFIT1 IFIT2 IFIT3 IFITM3 IL1RN IL33 ISG15 ITGA2 LGMN MMP7 MX1 MX2 NOX1 OAS1 OAS2 OAS3 PLA2G2A PLA2G2D PLAT PLAUR PPARG PTGES REL RETNLB SCARB1 TIRAP TLR2 TNFRSF12A |
| T_cell_co-inhibition | BTLA C10orf54 CD160 CD244 CD274 CTLA4 HAVCR2 LAG3 LAIR1 TIGIT |
| T_cell_co-stimulation | CD2 CD226 CD27 CD28 CD40LG ICOS SLAMF1 TNFRSF18 TNFRSF25 TNFRSF4 TNFRSF8 TNFRSF9 TNFSF14 |
| Type_I_IFN_Reponse | DDX4 IFIT1 IFIT2 IFIT3 IRF7 ISG20 MX1 MX2 RSAD2 TNFSF10 |
| Type_II_IFN_Reponse | AHR GPR146 SELP |

**Table S4.** Clinical characteristics of CLL patients in validation cohort

|  |  | **Number (%)** | **ferroptosis-related prognostic score (FPS)** | | |
| --- | --- | --- | --- | --- | --- |
|  |  |  | **low risk (%)** | **high risk (%)** | ***P* ^1^ value** |
| Gender | | | | | |
|  | Male | 24 (66.7) | 17 (70.8) | 7 (29.2) | 1.000 |
|  | Female | 12 (33.3) | 8 (66.7) | 4 (33.3) |  |
| Age | | | | | |
|  | ≤65 years | 23 (63.9) | 16 (69.6) | 7 (30.4) | 1.000 |
|  | >65 years | 13 (36.1) | 9 (69.2) | 4 (30.8) |  |
| Binet Stage | | | | | |
|  | A | 14 (38.8) | 11 (78.6) | 3 (21.4) | 0.467 |
|  | B or C | 22 (61.2) | 14 (63.6) | 8 (36.4) |  |
| ALC | | | | | |
|  | ≤50×10^9^/L | 20 (60.6) | 15 (75.0) | 5 (25.0) | 0.270 |
|  | >50×10^9^/L | 13 (39.4) | 7 (53.8) | 6 (46.2) |  |
| Hb | | | | | |
|  | <100 g/L | 27 (77.1) | 20 (74.1) | 7 (25.9) | 0.226 |
|  | >100 g/L | 8 (22.9) | 4 (50.0) | 4 (50.0) |  |
| Plt | | | | | |
|  | <100×10^9^/L | 28 (80.0) | 19 (67.9) | 9 (32.1) | 1.000 |
|  | >100×10^9^/L | 7 (20.0) | 5 (71.4) | 2 (28.6) |  |
| LDH | | | | | |
|  | ≤271 U/L | 25 (78.1) | 16 (64.0) | 9 (36.0) | 1.000 |
|  | >271 U/L | 7 (21.9) | 5 (71.4) | 2 (28.6) |  |
| β2-MG | | | | | |
|  | ≤3.5 mg/L | 22 (61.1) | 18 (81.8) | 4 (18.2) | 0.067 |
|  | >3.5 mg/L | 14 (38.9) | 7 (50.0) | 7 (50.0) |  |
| TP53 disruption | | | | | |
|  | Yes | 6 (16.7) | 3 (50.0) | 3 (50.0) | 0.343 |
|  | No | 30 (83.3) | 22 (73.3) | 8 (26.7) |  |
| IGHV mutation | | | | | |
|  | Yes | 27 (75.0) | 21 (77.8) | 6 (22.2) | 0.096 |
|  | No | 9 (25.0) | 4 (44.4) | 5 (55.6) |  |
| CLL-IPI | | | | | |
|  | 0−3 | 24 (66.7) | 19 (79.2) | 5 (20.8) | 0.124 |
|  | 4−10 | 12 (33.3) | 6 (50.0) | 6 (50.0) |  |

Abbreviations: ALC, absolute lymphocytic count; β2‐MG, β2‐microglobulin; Hb, hemoglobin; IGHV, immunoglobulin heavy chain variable region; IPI, international prognostic index; LDH, lactate dehydrogenase; Plt, platelet.

^1^ *P* value is according to chi-square test.
