# Supplementary figures and images for: Identifying a novel ferroptosis-related prognostic score for predicting prognosis in chronic lymphocytic leukemia
Source: Front Immunol. 2022 Oct 6;13:962000. doi: 10.3389/fimmu.2022.962000 (PMC9582233; doi:10.3389/fimmu.2022.962000)

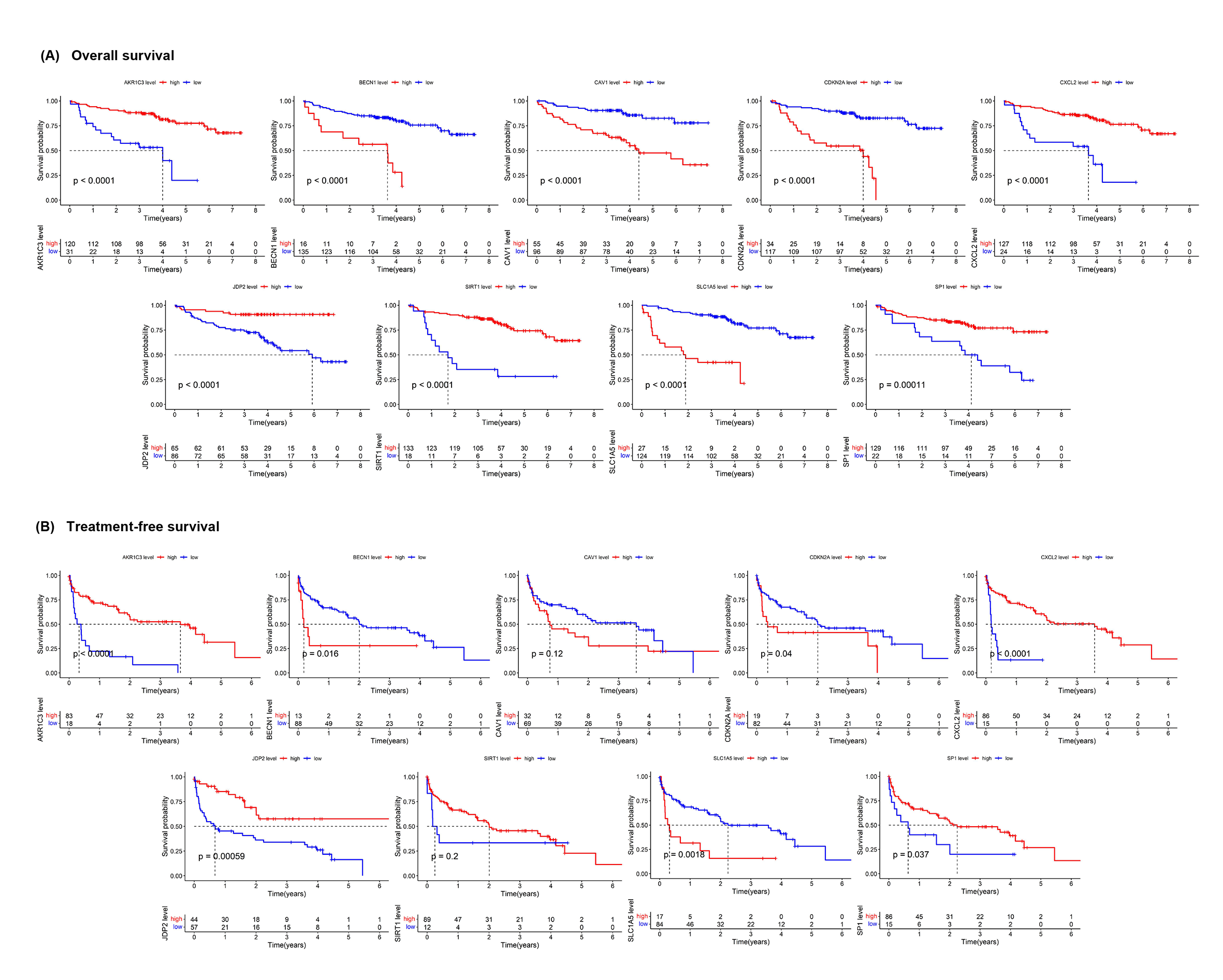

Supplement: Supplementary file 2 [file Image_1.tif]

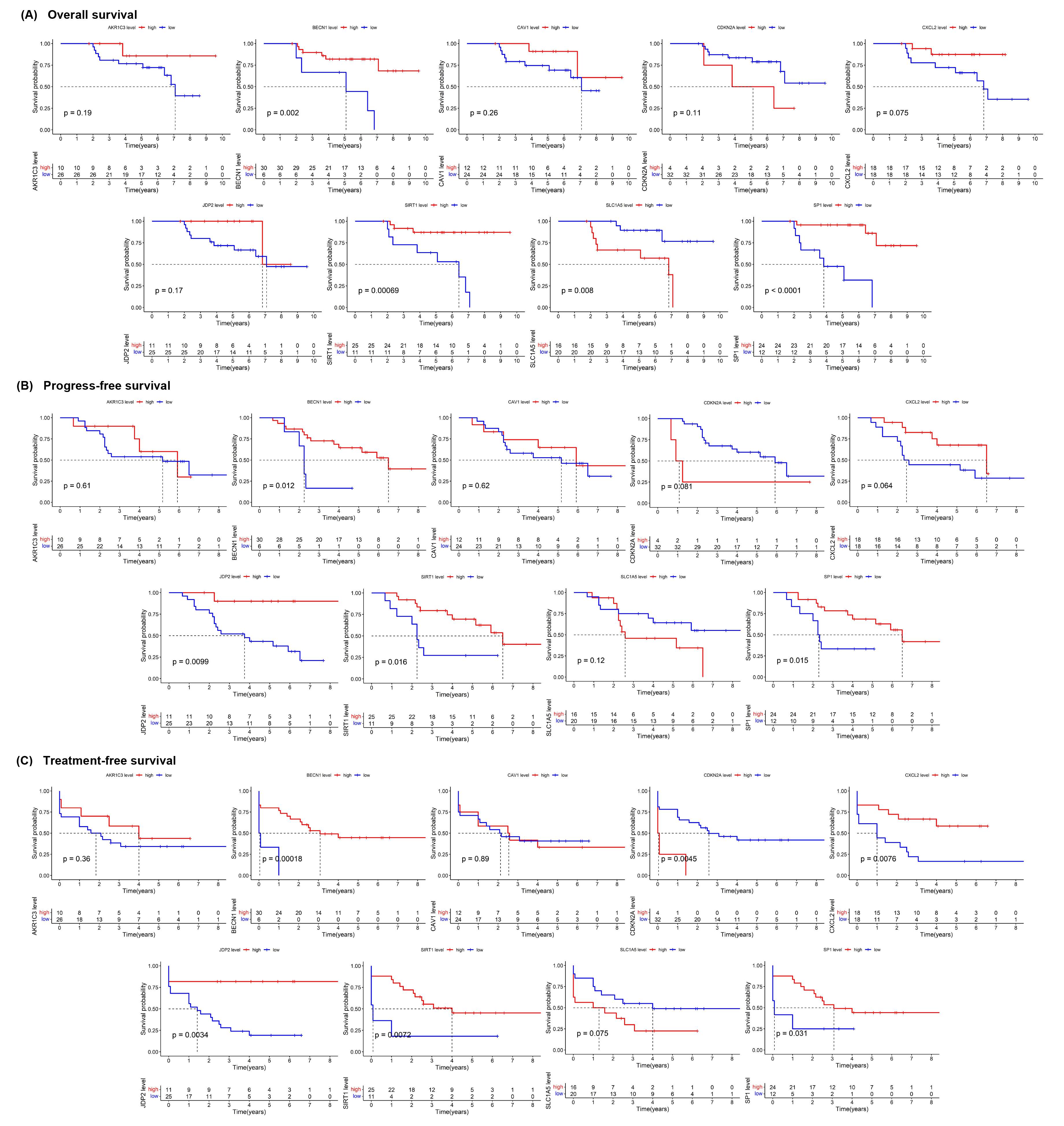

Supplement: Supplementary file 3 [file Image_2.tif]
